# The circadian E3 ligase FBXL21 regulates myoblast differentiation and sarcomere architecture via MYOZ1 ubiquitination and NFAT signaling

**DOI:** 10.1371/journal.pgen.1010574

**Published:** 2022-12-27

**Authors:** Ji Ye Lim, Eunju Kim, Collin M. Douglas, Marvin Wirianto, Chorong Han, Kaori Ono, Sun Young Kim, Justin H. Ji, Celia K. Tran, Zheng Chen, Karyn A. Esser, Seung-Hee Yoo

**Affiliations:** 1 Department of Biochemistry and Molecular Biology, The University of Texas Health Science Center at Houston, Houston, Texas, United States of America; 2 Department of Physiology and Functional Genomics, University of Florida College of Medicine, Gainesville, Florida, United States of America; The Jackson Laboratory, UNITED STATES

## Abstract

Numerous molecular and physiological processes in the skeletal muscle undergo circadian time-dependent oscillations in accordance with daily activity/rest cycles. The circadian regulatory mechanisms underlying these cyclic processes, especially at the post-transcriptional level, are not well defined. Previously, we reported that the circadian E3 ligase FBXL21 mediates rhythmic degradation of the sarcomere protein TCAP in conjunction with GSK-3β, and *Psttm* mice harboring an *Fbxl21* hypomorph allele show reduced muscle fiber diameter and impaired muscle function. To further elucidate the regulatory function of FBXL21 in skeletal muscle, we investigated another sarcomere protein, Myozenin1 (MYOZ1), that we identified as an FBXL21-binding protein from yeast 2-hybrid screening. We show that FBXL21 binding to MYOZ1 led to ubiquitination-mediated proteasomal degradation. GSK-3β co-expression and inhibition were found to accelerate and decelerate FBXL21-mediated MYOZ1 degradation, respectively. Previously, MYOZ1 has been shown to inhibit calcineurin/NFAT signaling important for muscle differentiation. In accordance, *Fbxl21* KO and *MyoZ1* KO in C2C12 cells impaired and enhanced myogenic differentiation respectively compared with control C2C12 cells, concomitant with distinct effects on NFAT nuclear localization and NFAT target gene expression. Importantly, in *Psttm* mice, both the levels and diurnal rhythm of NFAT2 nuclear localization were significantly diminished relative to wild-type mice, and circadian expression of NFAT target genes associated with muscle differentiation was also markedly dampened. Furthermore, *Psttm* mice exhibited significant disruption of sarcomere structure with a considerable excess of MYOZ1 accumulation in the Z-line. Taken together, our study illustrates a pivotal role of FBXL21 in sarcomere structure and muscle differentiation by regulating MYOZ1 degradation and NFAT2 signaling.

## Author summary

Skeletal muscle is a vital organ for our mobility, metabolism, and health. It is also highly dynamic at the molecular level, yet the underlying regulatory mechanisms are not well understood. Mounting evidence suggests a critical role of the biological timer, called circadian clock, in the molecular flux during the daily activity/rest cycle. Our previous research has revealed a circadian E3 ubiquitin ligase, FBXL21, as a regulator in the skeletal muscle. In the current study, we provide molecular and genetic evidence that FBXL21, with its upstream regulator GSK-3β, regulates degradation of a sarcomere protein, MYOZ1. Consistent with a key regulatory function, FBXL21 deficiency in *Psttm* mice and C2C12 cells led to marked abnormalities in muscle differentiation and function. Mechanistically, we show that FBXL21 and MYOZ1 regulate diurnal NFAT nuclear translocation and activity, a pathway important for muscle differentiation and development. Together, our work establishes a critical role of the circadian E3 ligase FBXL21 in muscle differentiation and sarcomere structure by regulating MYOZ1 and NFAT signaling.

## Introduction

Daily rhythms of physiology and behavior are controlled by our endogenous circadian clock [1–4]. In the mammalian clock system, the cellular oscillator consists of interlocked feedback loops containing positive (CLOCK/BMAL1, RORs) and negative (PERIOD1/2, CRYPTOCHROME1/2, and REV-ERBs) components, driving expression of clock-controlled genes (CCGs) and therefore governing tissue-specific and systemic functions throughout the body [2, 5–7]. Collective research has established the prevalence and complexity of circadian function and regulation. The vast majority of genes display oscillatory expression in at least one locale [5, 6], suggesting a near-ubiquitous circadian transcription regulatory circuit. In addition, growing evidence also underscores the pivotal role of post-transcriptional mechanisms, such as phosphorylation, acetylation, O-glycosylation, SUMOylation, and ubiquitination [8–11], in the circadian system. For example, proteomic analysis indicated that more than half of liver proteins showing circadian fluctuation in abundance do not correspond to oscillatory transcripts [12, 13]. Consistently, the core clock proteins are known to be directly regulated by the ubiquitination/proteasomal degradation pathway [14]. Our previous studies [15] illustrated that a pair of paralogous F-box-containing E3 ligases (FBXL3 and FBXL21) antagonistically regulate CRY proteins in a nucleus- and cytoplasm-specific manner, exemplifying an exquisite circadian post-translational control.

Skeletal muscle is an important target organ for the clock. Various aspects of skeletal muscle physiology are known to exhibit strong time-of-day-dependent oscillation, such as muscle contractile performance, glucose metabolism, muscle maintenance and growth, and structural organization [16–21]. Genetic and molecular studies have further revealed the regulatory function of the clock machinery in skeletal muscle structure and function [22, 23]. For example, a pioneering study showed that disruption of the positive arm of the oscillator, by either *Bmal1* deletion or *ClockΔ19* mutation, led to marked muscle fiber disorganization and impaired mitochondrial respiratory function [24]. More recently, we reported the identification of a new FBXL21 target protein, namely the sarcomere protein TCAP (titin cap) [19]. Mechanistic studies revealed GSK-3β functions as an upstream regulator of FBXL21 to promote circadian ubiquitination and degradation of TCAP [19]. Interestingly, physiological assays demonstrate significant skeletal muscle phenotypes in the *Psttm* mice harboring a hypomorph allele of *Fbxl21*, including reduced muscle fiber size and grip strength as well as impaired exercise tolerance in a circadian time-dependent manner [19]. This study illustrates FBXL21 as a key circadian regulator in the skeletal muscle.

Prompted by the observed skeletal muscle phenotypes in *Psttm* mice, our goal was to further investigate FBXL21 function in skeletal muscle and to explore FBXL21 deficiency as a genetic basis for modeling muscle dysfunction. In contrast to severe myopathies due to human TCAP mutations [25], TCAP knockout led to relatively mild phenotypes under normal conditions in mice [26], suggesting dysregulation of additional target proteins contributes to the marked muscle dysfunction in *Psttm* mice. Our yeast 2-hybrid (Y2H) screening identified Myozenin1 (MYOZ1; calsarcin-2) as a potential FBXL21 binding protein. MYOZ1 is a core Z-disc protein [27], believed to serve as a scaffold containing multiple binding sites for various sarcomere partners like α-actinin and TCAP to regulate skeletal muscle structure and function [27]. Importantly, MYOZ1 has been demonstrated to inhibit calcineurin-NFAT signaling by binding and sequestering calcineurin to Z-line, blocking NFAT nuclear translocation and transcriptional activation of target genes [27–29]. Given the central role of NFAT signaling in muscle differentiation [30–32], it would be interesting to interrogate the role of FBXL21-MYOZ1 in this process.

In the current study, we show that the GSK-3β-FBXL21 axis controls the proteasomal degradation of MYOZ1 by ubiquitination. We further illustrate the functional role of *Fbxl21* to regulate NFAT activation and myoblast differentiation. Our study therefore uncovers a novel FBXL21-mediated mechanism regulating MYOZ1-NFAT signaling and muscle differentiation and function.

## Results

### Identification of MYOZ1 as an FBXL21 target for ubiquitination-mediated degradation

**Fig 1:**
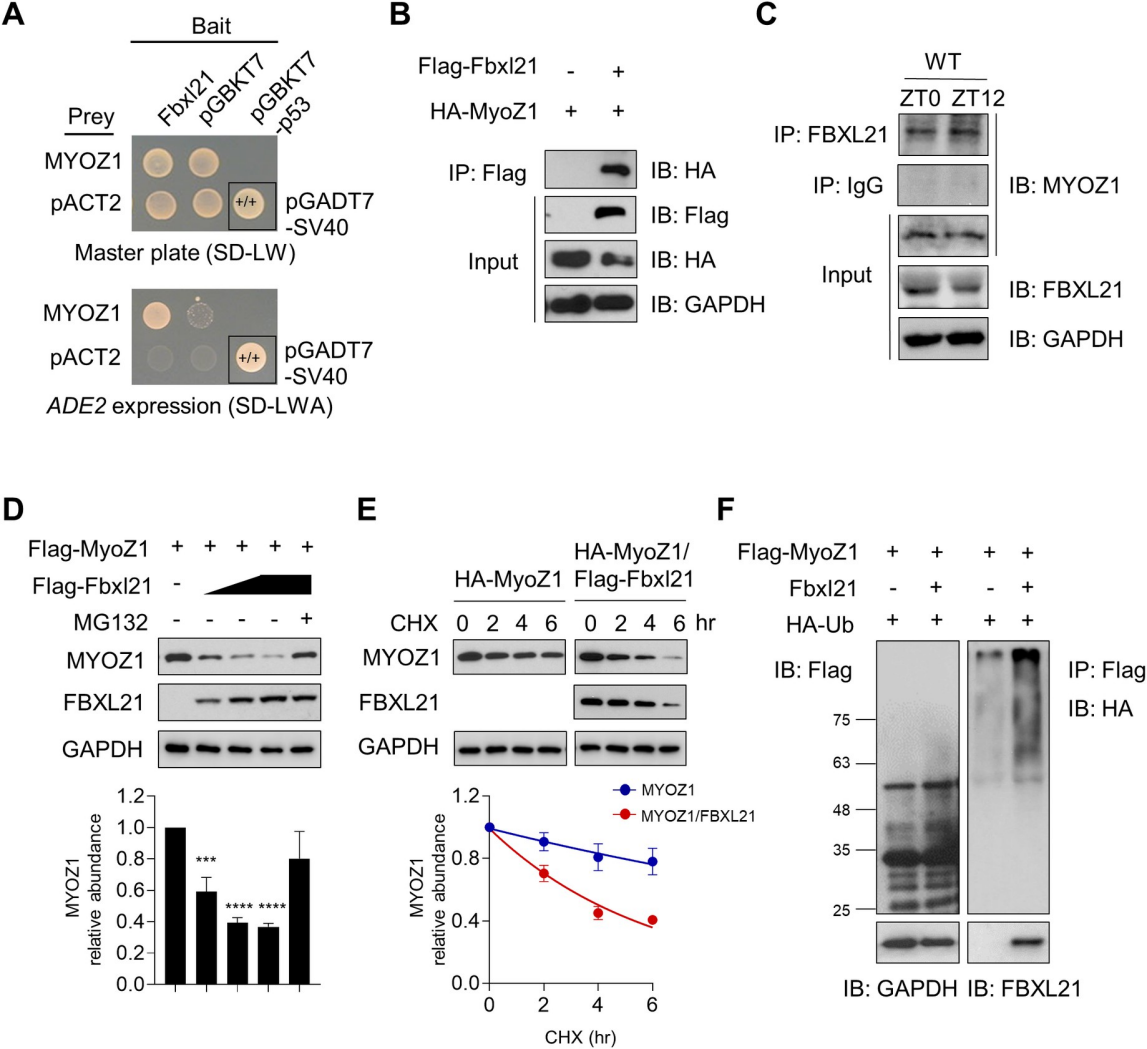
FBXL21 regulates MYOZ1 ubiquitination-mediated degradation. (A) Y2H test showing that FBXL21 interacts with MYOZ1. Positive control yeasts were transformed with pGBKT7-p53 (bait) and pGADT7-SV40 (prey) plasmids encoding the Gal4 DNA-binding domain (BD) fused with murine p53 and the Gal4 activation domain (AD) fused with SV40 large T antigen respectively. (B) Interaction of FBXL21 and MYOZ1. HEK 293T cells were transfected with Flag-Fbxl21 and HA-MyoZ1, and immunoprecipitation was performed using anti-FLAG antibody (M2). (C) Co-immunoprecipitation of MYOZ1/FBXL21 from gastrocnemius tissues of WT C57BL/6J mice at the indicated Zeitgeber time (ZT) using FBXL21 antibody. (D) FBXL21 expression decreased MYOZ1 amount in a dose-dependent manner. 293T cells were co-transfected with Flag-MyoZ1 with increasing amounts of Flag-Fbxl21 as indicated and treated with MG132. The quantification was done using unnormalized optical density (OD) values. Data are presented as mean ± SEM (n = 3). MYOZ1 abundance shows significant statistical differences between MYOZ1 alone and different FBXL21 concentrations. ***p < 0.001 and ****p < 0.0001; t-test. (E) MYOZ1 turnover was accelerated in the presence of FBXL21. 293T cells were co-transfected with the indicated plasmids before treatment with 100 μg/mL CHX. Immunoblotting was conducted to determine MYOZ1 and FBXL21 using anti-HA and anti-Flag antibodies. Lower panel: quantification of the effect of FBXL21 on MYOZ1 stability. Data are presented as mean ± SEM (n = 3). Half-life was calculated by using nonlinear, one-phase decay analysis by GraphPad Prism (MYOZ1, 15.8 hrs, MYOZ1/FBXL21, 4.1 hrs; the half-life parameter, K, is significantly different in Fbxl21 co-expression: ****p < 0.0001). (F) MYOZ1 ubiquitination mediated by FBXL21. Flag-MyoZ1 was co-transfected with plasmids expressing HA-tagged ubiquitin (HA-Ub), and Fbxl21 (no tag). The protein size markers are shown.

As reported previously, our Y2H screening identified the sarcomere protein TCAP as a FBXL21 binding protein [19]. Another hit from the same Y2H screen is MYOZ1 (Fig 1A), a sarcomere protein known to interact with TCAP. To biochemically validate MYOZ1 as a FBXL21-binding protein, we co-expressed MYOZ1 with FBXL21 in 293T cells, and co-immunoprecipitation (co-IP) assays showed FBXL21 interaction with MYOZ1 (Fig 1B). Interestingly, MYOZ1 displayed an anti-phasic circadian rhythm compared with FBXL21 in skeletal muscle (S1A Fig). To investigate their *in vivo* interaction, we performed co-IP using gastrocnemius tissues from wild-type (WT) C57BL/6 mice collected at ZT0 and ZT12 (ZT: Zeitgeber Time, with ZT0 and 12 corresponding to light on and off respectively) (Fig 1C). FBXL21 and MYOZ1 were found to display strong binding at both ZT0 and ZT12 (Fig 1C). We next investigated the effect of FBXL21 on MYOZ1 protein stability. FBXL21 ectopic expression in 293T cells reduced MYOZ1 protein level in a dose-dependent manner whereas the treatment of the 26S proteasome inhibitor MG132 enriched MYOZ1 (Fig 1D), suggesting that FBXL21 serves as an E3 ligase for MYOZ1 proteasomal degradation.

Next, we performed cycloheximide (CHX) chase assays to quantify the effect of FBXL21 on MYOZ1 turnover. The half-life of MYOZ1 protein was significantly decreased from 15.8 hrs to 4.1 hrs upon FBXL21 co-expression (Fig 1E), suggesting that FBXL21 accelerates MYOZ1 degradation in 293T cells. We carried out ubiquitination assays to examine whether FBXL21-mediated MYOZ1 degradation is ubiquitination-dependent. We expressed MYOZ1, FBXL21, and Ubiquitin (Ub) in 293T cells in the presence of the proteasome inhibitor MG132 (Fig 1F). FBXL21 co-expression strongly enriched poly-ubiquitinated MYOZ1 when compared to MYOZ1 expression by itself, suggesting FBXL21-mediated MYOZ1 ubiquitination (Fig 1F). Ectopic expression of MYOZ1 showed cytoplasmic accumulation and co-localization with FBXL21 (S1B Fig), indicating that MYOZ1 is a cytoplasmic substrate of FBXL21.

### The GSK-3β-FBXL21 axis regulates MYOZ1 degradation

**Fig 2:**
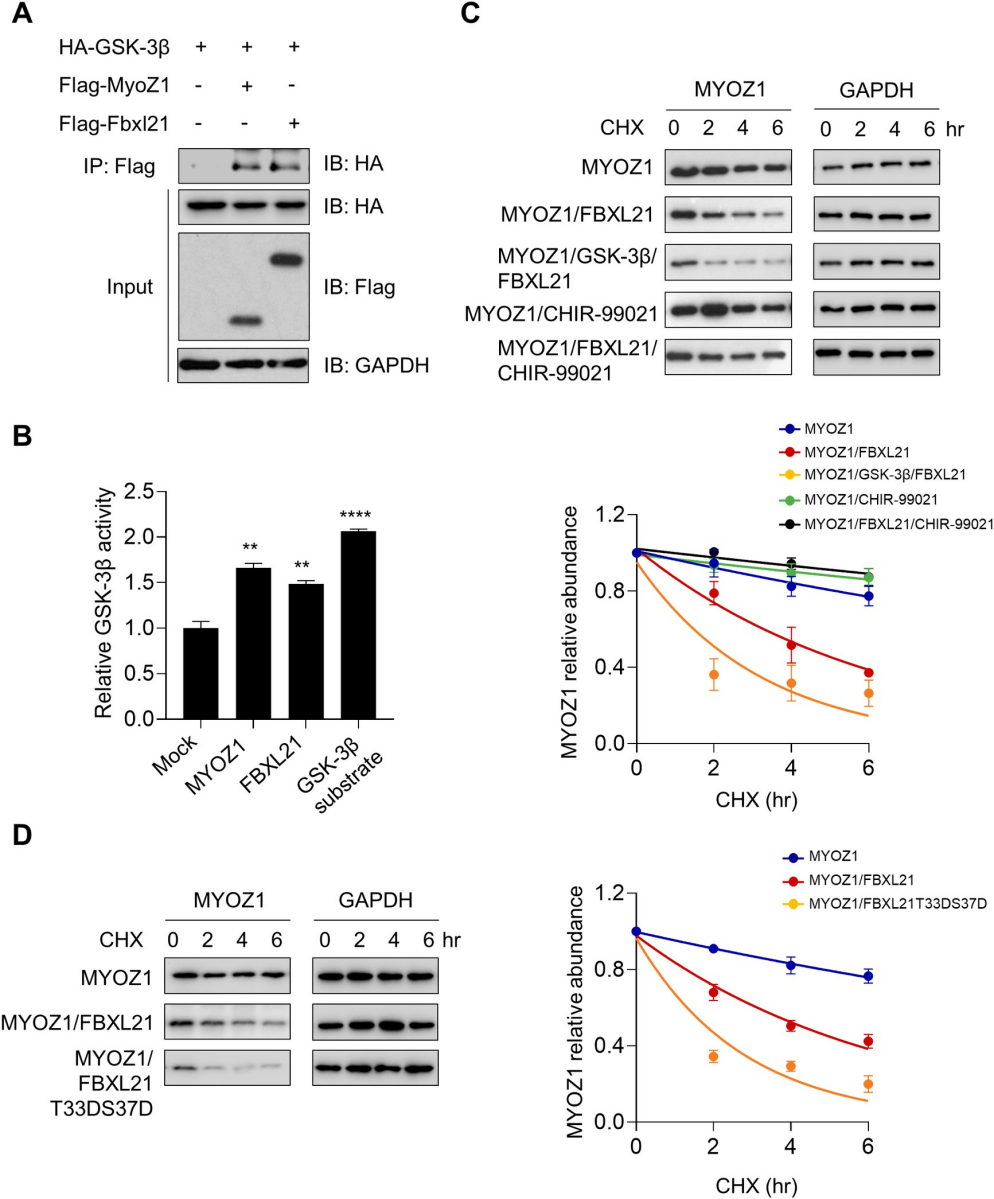
GSK-3β binds to MYOZ1 and regulates FBXL21-mediated MYOZ1 degradation. (A) Interaction of MYOZ1 and FBXL21 with GSK-3β. HEK 293T cells were co-transfected with the plasmids as indicated, and immunoprecipitation was conducted using anti-Flag antibody (M2). (B) GSK-3β *in vitro* kinase assay. Affinity purified Flag-MYOZ1 and Flag-FBXL21 from 293T cells, as well as a GSK-3β substrate peptide, were used as kinase reaction substrates. One-way ANOVA shows significant statistical differences between mock (pCMV-3XFlag empty vector) and Flag-MYOZ1, Flag-FBXL21 (**p < 0.01), and GSK-3β (****p < 0.0001). (C) The ectopic expression of GSK-3β and GSK-3 inhibitor CHIR-99021 accelerated and decelerated FBXL21-mediated MYOZ1 degradation, respectively. Lower panel: quantification of the effect of CHIR-99021 on MYOZ1 stability. Data are presented as mean ± SEM (n = 3). Half-life: MYOZ1, 15.4 h; MYOZ1/FBXL21, 4.3 h; MYOZ1/FBXL21/GSK-3β, 2.2 h; MYOZ1/CHIR-99021, 30.0 h; MYOZ1/FBXL21/CHIR-99021, 30.4 h. (D) The constitutively active FBXL21T33DS37D mutant showed enhanced E3 ligase activity for MYOZ1 degradation. Right panel: quantification of MYOZ1 degradation. Data are presented as mean ± SEM (n = 3). Half-life: MYOZ1, 15.2 h; MYOZ1/FBXL21, 4.4 h; MYOZ1/FBXL21T33DS37D, 1.9 h.

Our previous study showed that GSK-3β-FBXL21 regulates TCAP degradation via co-phosphorylation of FBXL21 and TCAP by GSK-3β [19]. To determine whether degradation of MYOZ1 is similarly controlled by the GSK-3β-FBXL21 axis, GSK-3β was co-expressed with MYOZ1 or FBXL21. Co-IP experiments showed that MYOZ1 binds to GSK-3β (Fig 2A), suggesting MYOZ1 could be a GSK-3β substrate. We therefore conducted *in vitro* kinase assays using affinity-purified FLAG-MYOZ1 and FLAG-FBXL21, which showed that MYOZ1 and FBXL21 were efficiently phosphorylated by GSK-3β (Fig 2B) [19]. Furthermore, in CHX chase assays, ectopic GSK-3β expression accelerated MYOZ1 degradation in an FBXL21-dependent manner, providing another evidence for a role of GSK-3β-FBXL21 in MYOZ1 degradation (Figs 2C and S2A). On the other hand, FBXL21-mediated MYOZ1 degradation was significantly retarded by the GSK-3β inhibitor CHIR-99021 (Figs 2C and S2A). Next, we generated a constitutively active form of FBXL21 by aspartate substitution of the GSK-3β phosphorylation and priming sites (T33 and S37 respectively). FBXL21T33DS37D showed significantly enhanced E3 ligase activity for MYOZ1 degradation compared to WT FBXL21 (Figs 2D and S2B). These results, together with our previous study [19], highlight a co-phosphorylation mechanism of FBXL21 and its target by GSK-3β underlying FBXL21-mediated substrate degradation.

### *Fbxl21* KO leads to MYOZ1 accumulation and inhibits myoblast differentiation in C2C12 cells

**Fig 3:**
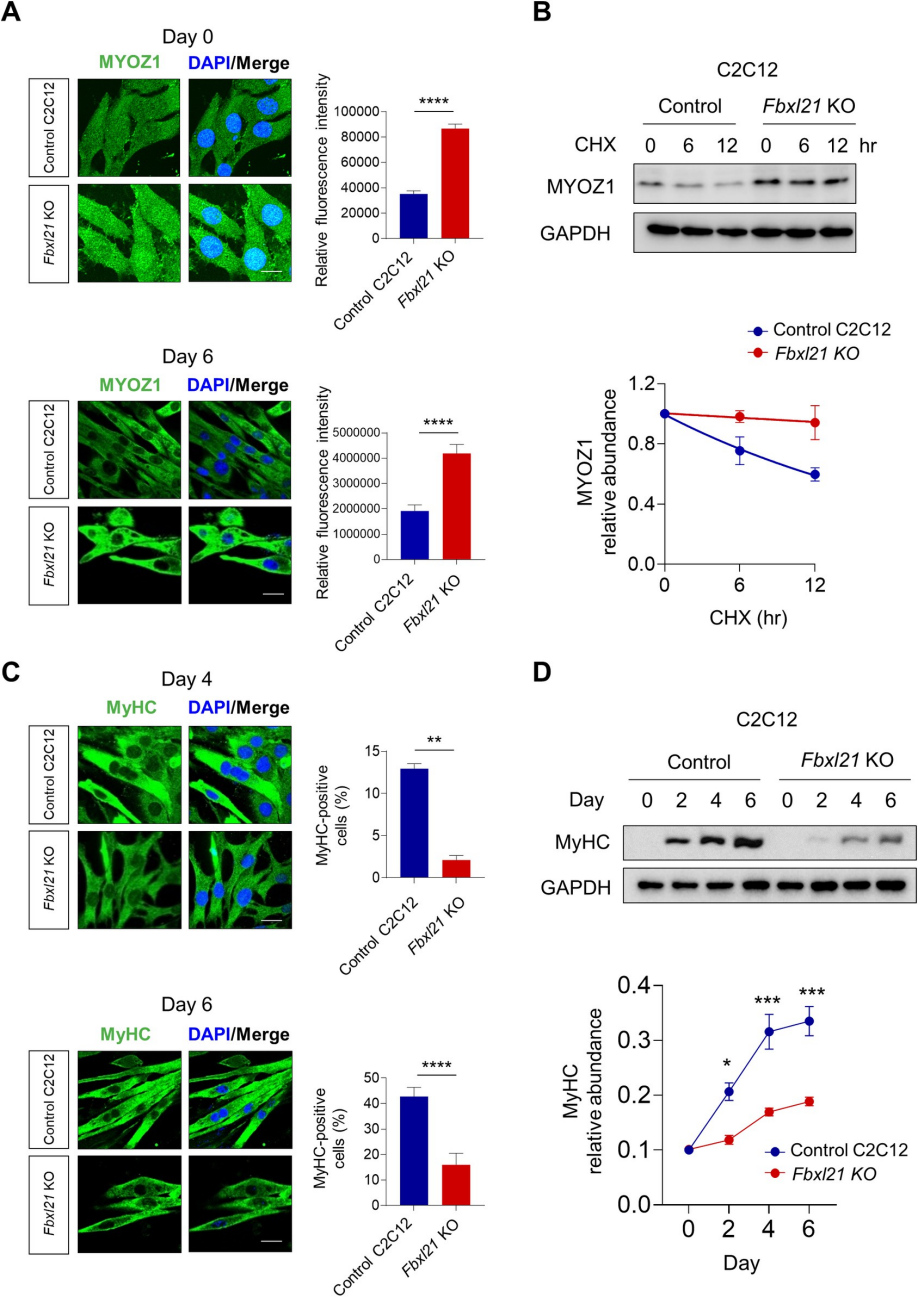
*Fbxl21* deletion leads to MYOZ1 accumulation and inhibits C2C12 myoblast differentiation. (A) MYOZ1 staining of control and *Fbxl21* KO C2C12 cells in myoblasts (differentiation Day 0) and myotubes (Day 6). Right panels: MYOZ1 fluorescence intensity quantification at day 0 and day 6. Data are presented as mean ± SEM (n = 3), ****p < 0.0001; t-test. control vs. *Fbxl21* KO C2C12 cells. Scale bar, 15 μm. (B) Analysis of MYOZ1 protein stability in control and *Fbxl21* KO C2C12 myoblasts by CHX chase assays. Lower panel: quantification of MYOZ1 stability. Half-life of MYOZ1: 15.8 hrs (control C2C12); > 24.0 hrs (*Fbxl21* KO C2C12). (C) MyHC staining of differentiated control and *Fbxl21* KO C2C12 cells (Day 4 and Day 6). Right panels: MyHC-positive cells (%) quantification (Day 4 and Day 6). Data are presented as mean ± SEM (n = 3), **p < 0.01 and ****p < 0.0001; t-test. control vs. *Fbxl21* KO C2C12 cells. Scale bar, 15 μm. (D) Immunoblotting analysis of MyHC protein expression in control and *Fbxl21* KO C2C12 cells during differentiation. GAPDH was used as a loading control. Lower panel: quantification of MyHC levels. Data are presented as mean ± SEM (n = 3), *p < 0.05 and ***p < 0.001; two-way ANOVA with Tukey’s multiple comparisons indicating a significant difference between control and *Fbxl21* KO C2C12 cells.

To investigate the effect of *Fbxl21* deletion on endogenous MYOZ1 levels in skeletal muscle cells, we used *Fbxl21* KO C2C12 cells (S3A Fig) previously generated by CRISPR-Cas9 [19] and performed immunofluorescence staining. *Fbxl21* KO and control C2C12 cells were differentiated to myotube for 6 days using DMEM supplemented with 2% horse serum. MYOZ1 levels were increased in myoblasts (Day 0) and myotubes (Day 6) of *Fbxl21* KO by 2.5 and 2.2 folds respectively compared to control C2C12 cells (Figs 3A and S3B). Next, we performed CHX chase assays using control and *Fbxl21* KO C2C12 cells, and observed that MYOZ1 degradation was significantly decelerated in *Fbxl21* KO (Fig 3B). To test whether *Fbxl21* KO and subsequent MYOZ1 accumulation affects myoblast differentiation, we stained control and *Fbxl21* KO C2C12 cells to monitor myosin heavy chain (MyHC), a muscle-specific terminal differentiation marker [33]. Consistent with reduced fiber diameters previously reported [19], MyHC expression was significantly lower in *Fbxl21* KO C2C12 cells compared to control C2C12 during differentiation (Day 4 and 6) (Fig 3C). At differentiation days 4 and 6, the fusion index (%), a well-established marker for myoblast differentiation [34, 35], was strongly reduced in *Fbxl21* KO C2C12 cells relative to control C2C12 cells (S3C Fig). Furthermore, immunoblotting analysis of MyHC indicated impaired differentiation of *Fbxl21* KO C2C12 cells compared to control cells (Fig 3D). These results demonstrate that *Fbxl21* deficiency leads to MYOZ1 accumulation and impairs C2C12 myoblast differentiation.

### Altered NFAT nuclear translocation in *Fbxl21* KO and *MyoZ1* KO C2C12 cells

The NFAT signaling pathway plays an important role in skeletal muscle differentiation [31, 36, 37]. It has been reported that MYOZ1 inhibits calcineurin/NFAT activity in C2C12 cells and skeletal muscle tissues [38]. To further investigate a direct link between MYOZ1 and NFAT2 signaling, we generated *MyoZ1* KO C2C12 myoblast cells by CRISPR-Cas9. qPCR and immunofluorescence staining showed that MyoZ1 was depleted in *MyoZ1* KO C2C12 cells (S4A and S4B Fig). After control and *MyoZ1* KO C2C12 cells were treated with the calcineurin stimulators PMA (20 ng/ml) and ionomycin (0.25 μM) for 1 hr, NFAT2 nuclear localization was measured by immunofluorescence staining. NFAT2 was mainly localized in the cytoplasm in both control and *MyoZ1* KO C2C12 cells treated with vehicle only (DMSO) (S4C Fig). Consistent with an inhibitory role of MYOZ1 for NFAT signaling, NFAT2 nuclear localization was much elevated in *MyoZ1* KO cells when compared to control C2C12 cells following the PMA and ionomycin treatment (S4C Fig). When control and *MyoZ1* KO C2C12 cells were induced for myogenic differentiation, MyHC-positive cells (%) and fusion index (%) were significantly higher in *MyoZ1* KO C2C12 cells when compared to control C2C12 cells at differentiation day 4 (S4D Fig). These results suggest that MYOZ1 regulates NFAT2 signaling and myoblast differentiation.

**Fig 4:**
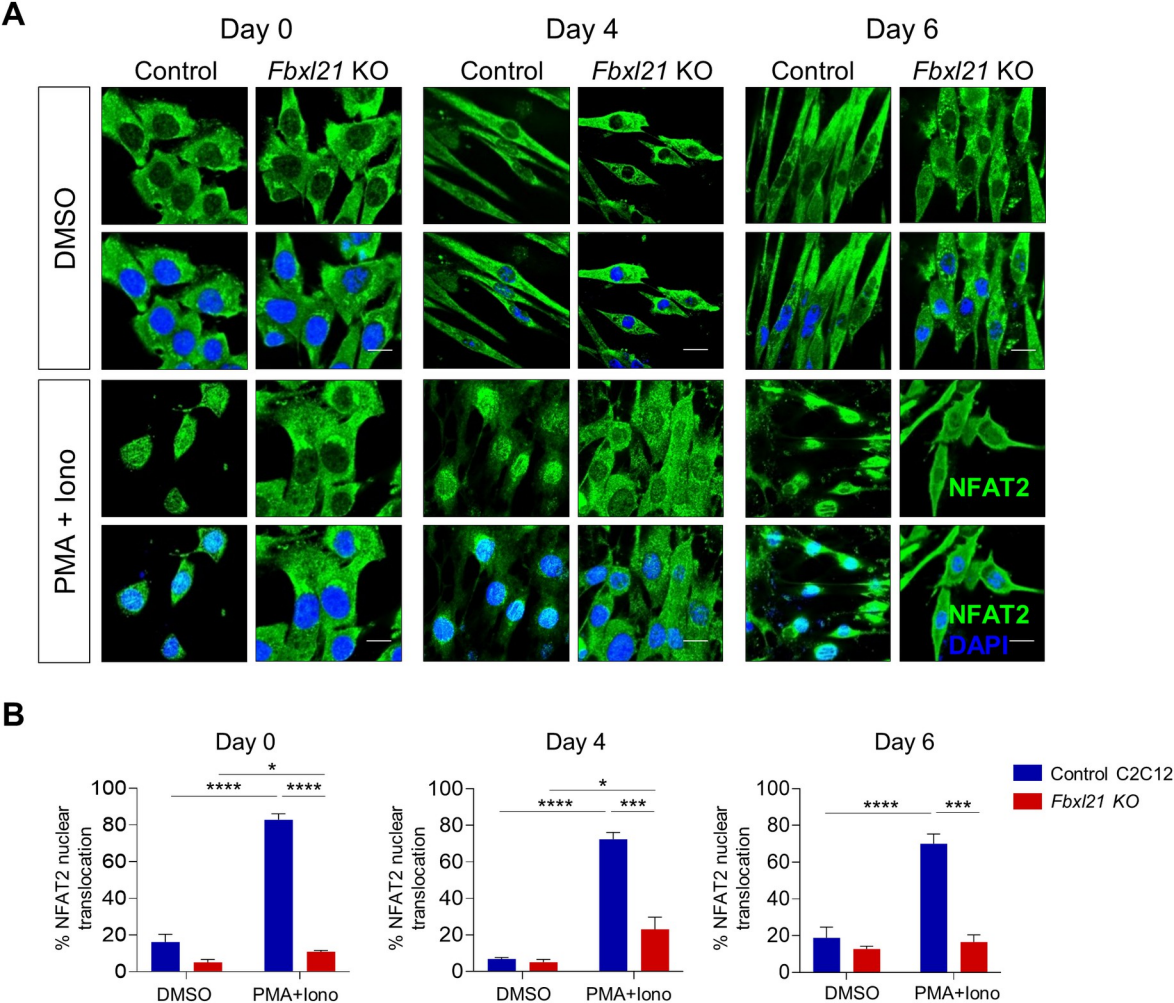
*Fbxl21* deletion impairs NFAT2 nuclear translocation in C2C12 cells. (A) Immunofluorescence staining of NFAT2 in control and *Fbxl21* KO C2C12 cells. Cells were differentiated for indicated days (Day 0, 4, and 6) with 2% horse serum, and treated with DMSO or the calcineurin activator PMA (20 ng/ml) and ionomycin (0.25 μM) (PMA + Iono) for 1 h. Control and *Fbxl21* KO C2C12 cells were immunostained with NFAT2 antibody, and imaged by confocal microscopy. Upon the treatment of PMA and ionomycin (PMA + Iono), NFAT2 nuclear translocation was significantly increased in control C2C12 cells at all differentiation stages (Day 0, 4, and 6), but was inhibited in *Fbxl21* KO C2C12 cells. (B) Quantification of NFAT2 nuclear translocation in control and *Fbxl21* KO C2C12 cells. Data are presented as mean ± SEM (n = 3), *p < 0.05, ***p < 0.001, and ****p < 0.0001; t-test. Scale bar, 15 μm.

Given that *Fbxl21* deletion induces MYOZ1 accumulation, we next examined whether *Fbxl21* deletion inhibits NFAT signaling. Following DMSO treatment, NFAT2 was mainly localized in the cytoplasm in both control and *Fbxl21* KO C2C12 cells (Fig 4A and 4B). Upon PMA and ionomycin treatment, NFAT2 nuclear translocation was significantly increased in control C2C12 cells while markedly reduced in *Fbxl21* KO C2C12 cells (Fig 4A and 4B). In addition, NFAT1 nuclear translocation upon PMA and ionomycin treatment was also significantly inhibited in *Fbxl21* KO C2C12 cells compared to control C2C12 cells (S4E Fig). Taken together, these data suggest that FBXL21-mediated MYOZ1 degradation regulates NFAT signaling.

**Fig 5:**
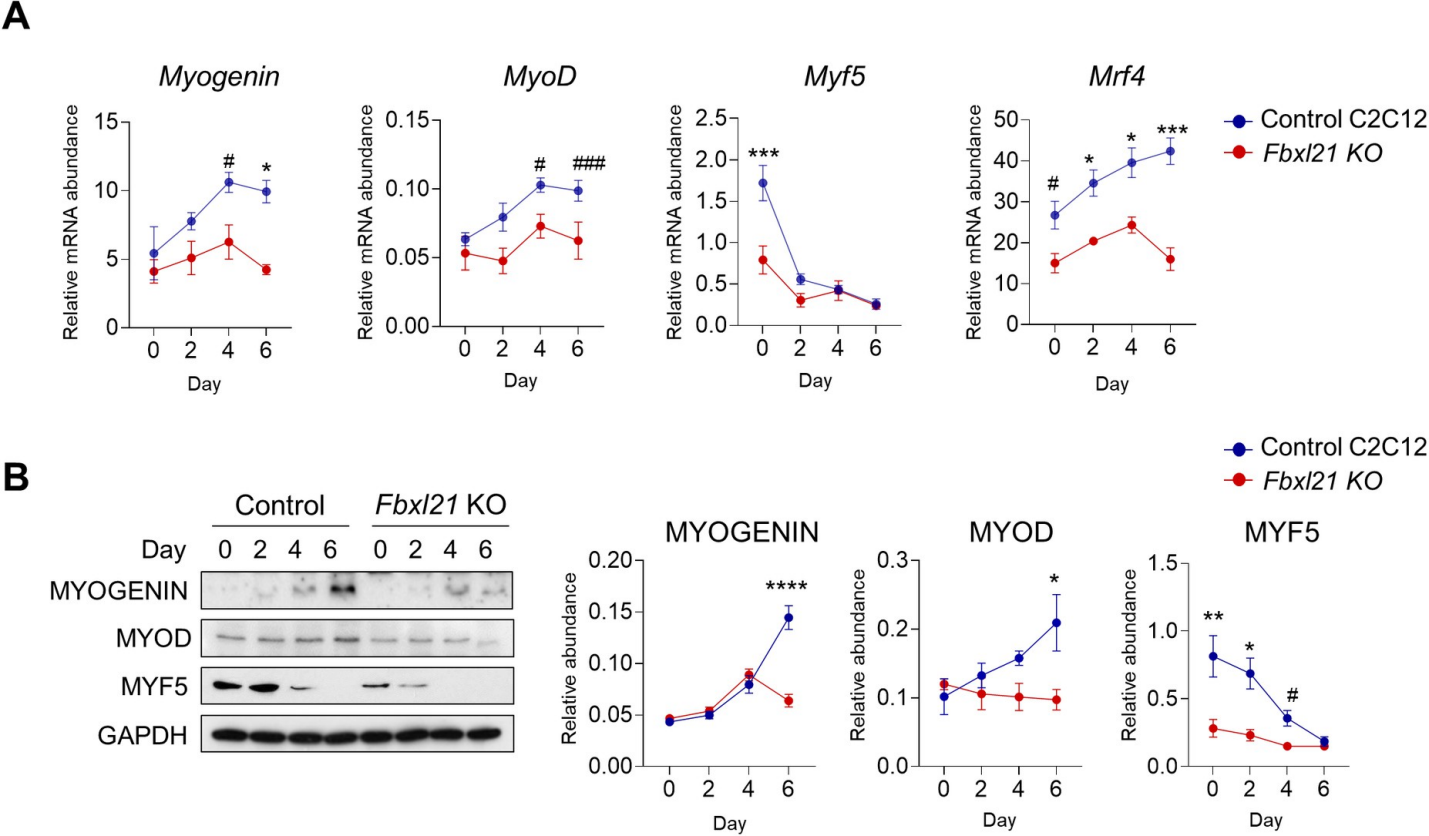
Expression of NFAT2 target genes was inhibited during myoblast differentiation in *Fbxl21* KO C2C12 cells. (A) Real-time RT-PCR analysis of NFAT target gene expression in control and *Fbxl21* KO C2C12 cells. *β-Actin* was used as internal control. Data are presented as mean ± SEM (n = 3), *p < 0.05, and ***p < 0.001; two-way ANOVA with Tukey’s multiple comparisons indicating significant difference between control and *Fbxl21* KO C2C12 cells. ^#^p < 0.05 and ^###^p < 0.001; t-test showing significant difference between control and *Fbxl21* KO C2C12 cells. (B) Immunoblotting analysis was performed to assess protein levels of MYOGENIN, MYOD, and MYF5 in control and *Fbxl21* KO C2C12 cells during differentiation. GAPDH was used as a loading control. Data are presented as mean ± SEM (n = 3), *p < 0.05, **p < 0.01, and ****p < 0.0001; two-way ANOVA with Tukey’s multiple comparisons indicating significant difference between control and *Fbxl21* KO C2C12 cells. ^#^p < 0.05; t-test showing significant difference between control and *Fbxl21* KO C2C12 cells.

### *Fbxl21* KO blocks NFAT target gene expression during myoblast differentiation

NFAT controls the expression of myogenic regulatory factor genes including *MyoD*, *Myf5*, *Myogenin*, and *Mrf4* [39–41]. To investigate the effects of *Fbxl21* deletion on NFAT target gene expression, *Fbxl21* KO C2C12 cells were differentiated to myotubes for 6 days, and mRNA and protein expressions of NFAT target genes were examined by qPCR and immunoblotting respectively. In control cells, we observed gradual increases (~2 fold) in mRNA levels of *MyoD*, *Myogenin*, and *Mrf4* genes during differentiation, whereas *Myf5* mRNA levels decreased sharply (Figs 5A and S5). In contrast, the overall mRNA abundance for these genes was strongly diminished in *Fbxl21* KO C2C12 cells (Figs 5A and S5). Consistent with the qPCR results, MYOGENIN and MYOD protein levels steadily increased during differentiation, while MYF5 protein amounts rapidly declined in control C2C12 cells (Figs 5B and S5). Abundance of these proteins was generally dampened in *Fbxl21* KO C2C12 cells (Figs 5B and S5). Our results suggest that FBXL21 plays a significant role in regulating NFAT target gene expression during myoblast differentiation.

**Fig 6:**
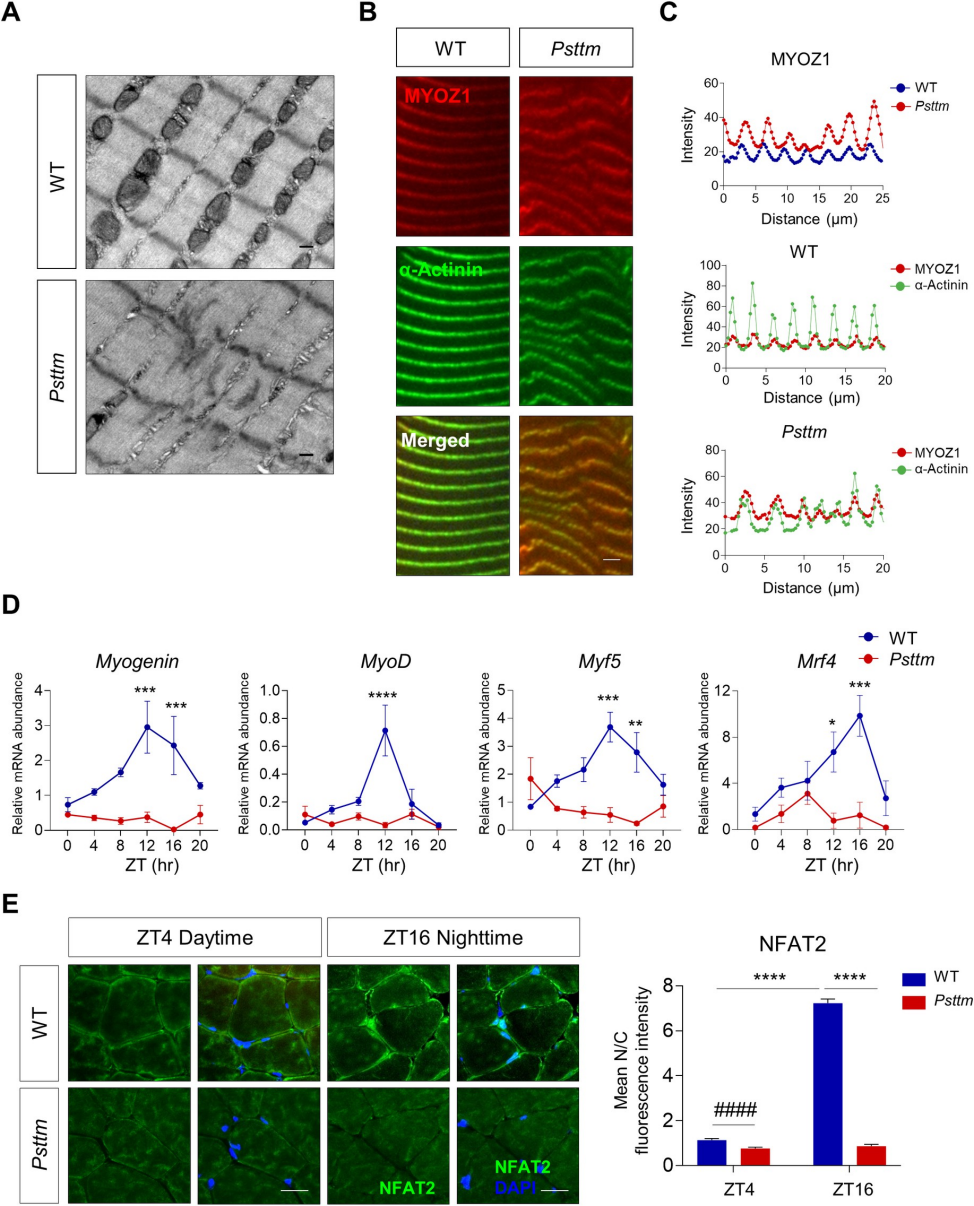
The *Fbxl21* hypomorph *Psttm* mice exhibited abnormal sarcomere structure, exaggerated MYOZ1 accumulation in the Z-line and impaired circadian regulation of calcineurin/NFAT signaling. (A) Transmission electron microscopy (TEM) of soleus tissues of WT and *Psttm* mice showing sarcomere abnormalities in *Psttm* mice. Scale bars, 0.2 μm. (B) Longitudinal gastrocnemius cryosections from WT and *Psttm* mice (n = 3 mice/group) were immunostained with MYOZ1 and α-actinin antibodies, and imaged by confocal microscopy. Scale bars, 2.5 μm. (C) Quantification of immunofluorescence images shows MYOZ1 accumulation in Z-line of *Psttm* mice compare to WT. Line profiles of the mean fluorescence intensities of MYOZ1 (red) and α-actinin (green) in Z-lines of WT and *Psttm* mice were obtained using Prism. (D) Real-time RT-PCR analysis of NFAT target expression in gastrocnemius tissues of WT and *Psttm* mice. Blue and red circles represent WT and *Psttm* mice, respectively. Data are presented as mean ± SEM (n = 3–4 mice/group/time point). *p < 0.05, **p < 0.01, ***p < 0.001, and ****p < 0.0001; two-way ANOVA with Tukey’s multiple comparisons indicating significant difference between WT and *Psttm* mice. JTK analysis showed that *Myogenin*, *MyoD*, *and Myf5* are rhythmic in WT mice, but not in *Psttm* mice (JTK_Cycle, adjusted p < 0.001 for *Myogenin*, *MyoD*, and *Myf5*) (S1 Table). (E) Nucleocytoplasmic localization of NFAT2 in WT and *Psttm* mice collected at ZT4 (daytime) and ZT16 (nighttime). Cross sections of gastrocnemius tissues from WT and *Psttm* mice (n = 5–6 mice /group/time point) were immunostained with NFAT2 antibody and DAPI. Right panel: Quantification of mean nuclear/cytoplasmic fluorescence intensity in more than 100 muscle fibers of WT and *Psttm* mice collected at ZT4 and ZT16. Data are presented as mean ± SEM (n = 5–6 mice/group/time point). ****p < 0.0001; two-way ANOVA with Tukey’s multiple comparisons indicating a significant difference between WT and *Psttm* mice or between two time points (ZT4 and ZT16) in WT mice. ^####^p < 0.0001; t-test showing significant difference between WT and *Psttm* mice in ZT4.

### *Fbxl21* is required for normal striated sarcomere pattern and circadian regulation of NFAT signaling *in vivo*

Myogenic differentiation is a complex process encompassing extensive alterations in subcellular architecture and cell morphology including sarcomere assembly, and highly organized sarcomeres are essential for maintaining muscle function [42–44]. Although we previously reported muscle function impairments in *Psttm* mice [19], sarcomere structure in these mice was not investigated. We therefore performed transmission electron microscopy (TEM) using soleus tissues from *Psttm* and WT littermates. At 6 months, in contrast to the normal, intact Z-line in WT mice, *Psttm* mice exhibited impaired sarcomere structure, including Z-line streaming (disrupted Z-line borders) [45] and indistinct A-bands (Fig 6A). This result indicates an important role of FBXL21 in maintaining sarcomere structure.

To investigate the role of *Fbxl21* in MYOZ1 regulation *in vivo*, we performed immunofluorescence staining with antibodies against MYOZ1 and α-actinin (a Z-line marker) using gastrocnemius tissues. MYOZ1 showed extensive overlapping immunofluorescence signals with α-actinin in both WT and *Psttm* tissues (Fig 6B and 6C). Interestingly, the MYOZ1 signal was significantly increased in *Psttm* compared to WT (Fig 6B and 6C). and Z-line irregularities were apparent, consistent with the Z-line streaming in the EM images.

Finally, we examined the role of FBXL21 in NFAT signaling *in vivo*. Previously, it has been demonstrated that NFAT nuclear translocation is under circadian control [46]. We therefore collected gastrocnemius muscles from WT and *Psttm* mice over the circadian cycle to investigate rhythmic expression of NFAT target genes. In WT mice, NFAT2 target genes (*Myogenin*, *MyoD*, and *Myf5*) showed highly oscillatory expression patterns, in contrast, both the expression level and oscillatory amplitude of these genes were significantly dampened in *Psttm* mice (Fig 6D and S1 Table). Next, we examined NFAT nuclear localization in gastrocnemius muscles by immunofluorescence staining. NFAT2 nuclear localization was markedly increased at ZT16 compared to ZT4 in WT muscles (Fig 6E), and the levels were inversely correlated with the low and high MYOZ1 expressions at these time points as shown in circadian immunoblotting (S1A Fig) and immunofluorescence staining (S6A Fig). In comparison, *Psttm* muscles showed diminished levels of NFAT2 nuclear localization at both time points (more profoundly at ZT16) (Fig 6E) as well as a disrupted diurnal pattern of MYOZ1 expression (S6A Fig). Of note, the MYOZ1 accumulation in *Psttm* mice correlated with markedly attenuated amounts of FBXL21 compared with WT mice (S6B Fig). Collectively, our results indicate a key regulatory function of FBXL21 in sarcomere structure and NFAT signaling *in vivo*.

## Discussion

The ubiquitin-proteasome system (UPS) plays an important role in skeletal muscle physiology, dynamically modulating muscle mass, contractile properties, and metabolism [47]. Previously, we reported that the clock-controlled E3 ligase FBXL21 regulates circadian TCAP degradation and skeletal muscle function [19]. Here, we identified MYOZ1, a TCAP-binding protein in the sarcomere, as a new substrate for FBXL21-mediated ubiquitination and proteasomal degradation. Similar to FBXL21 ubiquitinating MYOZ1 and TCAP, another E3 ligase, the FANCL subunit of the FA (Fanconi anemia) core complex, has been shown to monoubiquitinate two interacting proteins, namely FANCD2 and FANCI [48, 49]. Our study further revealed that GSK-3β phosphorylates MYOZ1 and promotes its degradation, reminiscent of its action to co-phosphorylate FBXL21 and TCAP for TCAP turnover [19]. Consistent with the important role of calcineurin/NFAT signaling in muscle differentiation, we observed impaired and enhanced myogenic differentiation in *Fbxl21* KO and *MyoZ1* KO C2C12 cells respectively compared with control C2C12 cells, concomitant with distinct effects on NFAT nuclear localization and NFAT target gene expression. *In vivo* studies using *Psttm* mice further revealed that both the levels and diurnal rhythm of NFAT2 nuclear localization were significantly dampened, and circadian expression of NFAT target genes for muscle differentiation was also decreased compared with WT. Finally, *Psttm* mice exhibited significant disruption of sarcomere structure with exaggerated MYOZ1 accumulation in the Z-line. Extending our previous study [19], the current work highlights a regulatory function of GSK-3β-FBXL21 impinging on MYOZ1/NFAT signaling in myoblast differentiation and sarcomere organization, thereby governing diurnal skeletal muscle function.

Altered expression of sarcomere proteins can lead to disruption of Z-line architecture [50, 51]. MYOZ1 was identified as one of the calcineurin-interacting proteins (calsarcins), functioning to harness calcineurin to α-actinin at the Z-line [29]. A recent structural study [27] found that MYOZ1 (FATZ1) has multiple binding sites for other sarcomere proteins, creating an interaction hub for Z-line proteins [27]. For example, MYOZ1 binds to α-actinin-2/-3, myotilin, and filamin-C through its C-terminal region [27]. Of note, MYOZ1 forms a 2:1 complex with α-actinin-2 which stabilizes MYOZ1 proteins at the Z-disc [27]. Therefore, increased MYOZ1 levels as a result of FBXL21 deficiency may lead to the dysregulated stoichiometry of sarcomeric components, which in turn contributes to sarcomere structural alteration in *Psttm* mice.

In our study, multiple assays were employed to establish FBXL21 as an E3 ligase targeting MYOZ1. Previously the E3 ligases MuRF1 and MuRF2 were suggested to target and ubiquitinate MYOZ1 [52], and MuRF1/MuRF2 double KO mice showed altered myofiber alignment and abnormal mitochondrial ultrastructure in the myocardium [53]. However, MYOZ1 expression was largely ablated in MuRF1/2 double KO mice, suggesting that MuRF1/2 may not be the key E3 ligases acting to downregulate MYOZ1 by ubiquitination [52]. In our study, we provide biochemical and molecular evidence indicating that FBXL21 directly binds and ubiquitinates MYOZ1. We also show that MYOZ1 protein levels were elevated in *Fbxl21*-deficient C2C12 cells and *Psttm* mice, thereby inhibiting NFAT signaling. Similar to our results, increased MYOZ1 levels in conjunction with decreased MyHC were observed in muscle disuse conditions [54]. In comparison, MyoZ1 KO mice showed improved exercise performance and enhanced running distance by upregulating calcineurin/NFAT activity in their skeletal muscle tissues when compared to WT mice [38].

Here we show that NFAT nuclear translocation is diurnal, a process regulated by FBXL21 and MYOZ1; in accordance, NFAT target genes functioning in muscle differentiation display strong circadian oscillatory expression in WT mice, but not in *Psttm* mice. NFAT is a master transcription factor known to play a crucial role in myoblast differentiation [39–41], muscle fiber type switching [38, 55, 56], muscle regeneration [57], and hypertrophy [58]. When activated, NFAT translocates to the nucleus, followed by active nuclear export to shuttle back to the cytoplasm [59, 60]. Consistent with our results, NFAT nuclear translocation is subject to circadian control, driving rhythmic expression of target genes in the skeletal muscle [46, 61]. Furthermore, NFAT nuclear translocation was found to exhibit a daily rhythm in the left ventricle of mouse heart, which was abolished by calcineurin inhibitors [62]. Therefore, Ca^2+^-calcineurin-NFAT is a crucial pathway integrating external stimuli and circadian gene expression [46]. It has been shown that a small molecule targeting NFAT signaling perturbed PER1 and CRY1 nuclear translocation and affected circadian rhythms [63]. It is possible that rhythmic NFAT nuclear translocation directly drives the circadian oscillation of *MyoD*, *Myf5*, and *Myogenin* in WT mice, whereas increased MYOZ1 in *Psttm* mice inhibited NFAT and subsequently dampened target gene expression. In our previous study, we identified E-box elements for CLOCK/BMAL1 binding on the *MyoD* gene promoter [64], suggesting that both NFAT and CLOCK/BMAL1 synergistically regulate the circadian oscillation of *MyoD*. Likewise, previous ChIP-seq studies have revealed CLOCK or BMAL1 binding to *Myogenin* and *Mrf* gene promoters [65], indicating a coordinate regulation of these genes involving NFAT and circadian factors. While other transcription factors have also been reported to regulate these genes [66–70], it is interesting to note that the circadian peak of NFATc1 (also called NFAT2) nuclear translocation coincides with that of *Mrf4* transcript expression [46], suggesting close mechanistic crosstalk between NFAT and the clock machinery.

In conclusion, we identified the Z-line protein MYOZ1 as a new target of the circadian E3 ligase FBXL21, and FBXL21 deficiency led to Z-line disorganization, NFAT inhibition, and impaired muscle differentiation. Our study underscores a pivotal role of FBXL21 as a circadian E3 ligase in skeletal muscle, suggesting a new genetic and therapeutic target for muscle disorders.

## Materials and methods

### Animal studies and ethics statement

C57BL/6J (Stock # 000664) mice were purchased from the Jackson Laboratory (Bar Harbor, ME, USA); *Psttm* and littermate WT mice were maintained in-house. All mice were housed under LD12:12 unless otherwise noted. All animal studies, using male mice, were approved by The University of Texas Health Science Center (UTHealth Houston) Center for Laboratory Animal Medicine and Care (CLAMC).

### Cell culture, transfection and differentiation

293T (ATCC: CRL-3216) cells and C2C12 (ATCC: CRL-1772) were cultured in DMEM supplemented with 10% fetal bovine serum (GenDEPOT, Houston, TX, USA). For immunoprecipitations, 2 × 10^6^ cells were plated into 60 mm dishes 1 day before transfection, and iMFectin (GenDEPOT, Houston, TX, USA) was used to transfect DNA according to the manufacturer’s protocol. For C2C12 differentiation, cells were maintained in DMEM with 10% FBS and penicillin/streptomycin until 80–90% confluence. Cells were incubated in differentiation media (DMEM containing 2% horse serum and penicillin/streptomycin). Differentiation media were changed daily until cells were fully differentiated (around day 6). To quantify the fusion capacity of myoblasts indicative of differentiation potential, we calculated the fusion index as the percentage of nuclei in MyHC-positive myotubes with two or more nuclei in a given field [34, 35]. Fbxl21 CRISPR and MyoZ1 CRISPR cell lines were generated as described previously [19]. Briefly, the MyoZ1 guide RNA pairs (mMyoZ1(-) forward and reverse: CACCGGAACCCCGGCCCCTAACAAG, AAACCTTGTTAGGGGCCGGGGTTC; mMyoZ1(+) forward and reverse: CACCGCCTCCTGGATAAGGTGGCCG, and AAACCGGCCACCTTATCCAGGAGG) were designed (http://crispr.mit.edu/) and cloned into the BsmB1 site of the LentiCRISPR v2. For the control plasmid, no guide RNA pairs were inserted into the construct. C2C12 cells (ATCC CRL1772) were transfected with the LentiCRISPR v2 including guided RNA or control plasmid. Following transfection, cells were selected using puromycin (2 μg/ml). Individual clones were expanded and analyzed by qPCR or immunofluorescence to verify successful CRISPR knockout.

### Immunoblotting, immunoprecipitation, and immunocytochemistry

Immunoblotting, immunoprecipitation, and immunocytochemistry were performed as described previously [15, 19, 71, 72]. M2 agarose bead (Sigma-Aldrich, St Louis, MO, USA) was used to pull down flag-tagged proteins. MYOZ1 degradation assays were performed by transfecting pCMV10-3XFlag-MYOZ1 with or without Fbxl21 expression constructs into 2 × 10^5^ 293T cells in a 12-well plate. Thirty-six hours after transfection 100 μg/mL cycloheximide (CHX) was added and cells were collected at the indicated times. Half-life was determined by using a nonlinear, one-phase decay analysis (GraphPad Prism). CHIR-99021 was purchased from Selleckchem. To detect ectopic expression of Flag-Fbxl21, Flag-MyoZ1, HA-Gsk-3β and HA-MyoZ1, Flag-HRP (Sigma-Aldrich, St Louis, MO, USA) and HA antibodies (Roche, Hercules, CA, USA) were used. For endogenous MYOZ1 expression detection from skeletal muscle and C2C12 samples, anti-MYOZ1 antibody (Abcam, Cambridge, Maine, USA) was used. For endogenous MyHC and NFAT2 staining in C2C12 cells, anti-MyHC (R&D systems, Minneapolis, MN, USA) and NFAT antibodies (ABclonal Biotech, Cambridge, USA) were used. For FBXL21 immunoprecipitation, immunoblotting, and staining in C2C12 cells and muscle tissues, antibodies against FBXL21 were generated using rabbit (Cocalico Biologicals, Reamstown, PA, USA) as described previously [15] and serum was affinity purified using the same protein or peptide used to raise the antibody. For immunoblotting of differentiated C2C12 cells, commercial vendors of antibodies including MYOGENIN, MYOD, MYF5 (ABclonal Biotech, Cambridge, USA), α-actinin (Sigma-Aldrich, St Louis, MO, USA) and GAPDH (Abcam, Cambridge, Maine, USA) were used. Ubiquitination assays were performed as described before [15, 19]. For immunocytochemistry, negative controls were incubated with secondary antibodies only. The results are expressed as relative fluorescence intensity relative to the negative control (secondary antibody).

### *In vitro* kinase assay

Flag-MyoZ1 and Flag-Fbxl21 were ectopically expressed in 293T cells and affinity purified using M2 agarose bead (Sigma-Aldrich, St Louis, MO, USA), and cells transfected with the Flag-pCMV10 empty vector used as a mock control. These proteins were used as substrates for *in vitro* kinase assays by using GSK-3β Kinase Enzyme System with Z’-LYTE Kinase assay kit (Invitrogen, Carlsbad, CA, USA).

### RNA isolation and real-time qPCR

Total RNA was isolated from cells or frozen skeletal muscle tissue by using PureXtract RNAsol RNA isolation solution (GenDEPOT, Houston, TX, USA) as previously described [73]. Two micrograms of total RNA were used for cDNA synthesis (GenDEPOT, Houston, TX, USA). For PCR amplifications, amfiSure qGreen Q-PCR Master Mix (GenDEPOT, Houston, TX, USA) and QuantStudio 7 Real-time PCR machine were used. Relative mRNA abundance was measured using a comparative Ct method (ΔΔCt method) [74] to normalize mRNA levels to those of *Gapdh* or *β-actin*. The primer sequences are listed in S2 Table.

### Transmission electron microscopy

The soleus tissues were dissected from WT and *Psttm* mice age of 6 months, and immediately fixed with 3% glutaraldehyde, 4% paraformaldehyde in 0.1 M sodium cacodylate buffer (Na(CH_3_)_2_AsO_2_ •3H_2_O), pH 7.2, at 4°C. Samples were then post-fix with 2% osmium tetroxide for 1h. Then, samples were dehydrated in serial ethanol, and embedded in flat molds (5 LS, 1 XS). Ultra-thin sections (100 nm thick) were obtained by a Leica Ultracut-R microtome, and collected in copper grids (EMS). Sections were investigated in a JEOL 1200 transmission electron microscope equipped with a 2k x 2k Gatan CC camera. Images were acquired at the electron Cryo-microscopy core facility of the UTHealth McGovern Medical School.

### Quantification and statistical analysis

Results are presented as mean ± SEM unless otherwise stated. Statistical analyses were conducted using GraphPad Prism (version 9, GraphPad Software, Inc.) and JTK_Cycle nonparametric algorithm [75]. Data were analyzed using student’s t-test, one- or two-way ANOVA followed by Tukey’s post-hoc multiple comparison tests. Rhythmic patterns in gene expression were determined by JTK_Cycle. A value of p < 0.05 was considered statistically significant.

## Supporting information

S1 Fig Circadian rhythms and co-localization of MYOZ1 and FBXL21. (A) MYOZ1 and FBXL21 show anti-phasic circadian oscillations in skeletal muscle. Lower panels: quantification of MYOZ1 and FBXL21 levels. Data are presented as mean ± SEM (n = 3 mice/time point). *p < 0.05; Two-way ANOVA shows a statistical difference between MYOZ1 and FBXL21. One-way ANOVA with Tukey’s post hoc analysis shows a statically significant differences in MYOZ1 amount between time points (ZT4 vs ZT16: *p < 0.05, ZT4 vs ZT20: *p < 0.05). (B) Co-localization of MYOZ1 and FBXL21 in 293T cells. 293T cells were transfected with indicated plasmids, and immunofluorescence staining was performed using the indicated antibodies. Scale bars, 15 μm.
(PDF)

S2 Fig Immunoblotting analysis of FBXLs expression in 293T cells. (A) FBXL21 protein expression in Fig 2C. (B) Protein expression of WT FBXL21 and FBXL21T33DS37D mutant in 293T cells in Fig 2D. 293T cells were transfected with the indicated plasmids. Cells were incubated with 100 μg/mL CHX for indicated time before harvest.
(PDF)

S3 Fig Characterization and differentiation of *Fbxl21* KO C2C12 cells. (A) Loss of FBXL21 expression in *Fbxl21* KO C2C12 cells compared to control cells. Successful *Fbxl21* knockdown using the CRISPR-Cas9 was confirmed by immunofluorescence. Scale bars, 15 μm. (B) DAPI staining for Fig 3A. Scale bars, 15 μm. (C) Fusion index (%) quantification of Fig 3C. Control and *Fbxl21* KO C2C12 cells were induced to differentiate for the indicated days (day 4 and day 6). The fusion index (%) was calculated. Data are presented as mean ± SEM (n = 3), ****p < 0.0001; Two-way ANOVA shows a statistical difference between control and *Fbxl21* KO C2C12 cells.
(PDF)

S4 Fig Altered NFAT localization in *MyoZ1* KO and *Fbxl21* KO C2C12 cells. (A) qPCR of *MyoZ1* mRNA in control and *MyoZ1* KO C2C12 cells. Data are presented as mean ± SEM (n = 3), **p < 0.01; t-test. control vs. *MyoZ1* KO C2C12 cells. (B) Representative MYOZ1 immunofluorescence staining image of successful *MyoZ1* knockdown in C2C12 cells using CRISPR-Cas9. Scale bars, 15 μm. (C) Immunofluorescence staining of NFAT2 in control and *MyoZ1* KO C2C12 myoblast cells. Control and *MyoZ1* KO C2C12 myoblast cells were treated with DMSO or the calcineurin stimulators PMA (20 ng/ml) and ionomycin (0.25 μM) (PMA + Iono) for 1 hr. Right panel: quantification of % NFAT2 nuclear translocation. Data are presented as mean ± SEM (n = 4–6). Scale bars, 15 μm. ****p < 0.0001; t-test. Scale bars, 15 μm. (D) Immunofluorescence staining of MyHC in control and *MyoZ1* KO C2C12 cells. Control and *MyoZ1* KO C2C12 cells were differentiated for 4 days and stained with MyHC. Middle panel: MyHC-positive cells (%), right panel: fusion index (%) were calculated. Data are presented as mean ± SEM (n = 3). **p < 0.01 and ****p < 0.0001; t-test between control and *MyoZ1* KO C2C12 cells. (E) Control and *Fbxl21* KO C2C12 cells were treated with DMSO or the calcineurin stimulators PMA (20 ng/ml) and ionomycin (0.25 μM) (PMA + Iono) for 1 hr. Control and *Fbxl21* KO C2C12 cells were immunostained for NFAT1 and imaged by confocal microscopy. In response to PMA and ionomycin treatment, NFAT1 nuclear translocation was increased in control C2C12 cells, while markedly decreased in *Fbxl21* KO C2C12 cells. Right panel: quantification of NFAT1 nuclear translocation in control and *Fbxl21* KO C2C12 cells. Data are presented as mean ± SEM (n = 3), *p < 0.05 and ****p < 0.0001; t-test. Scale bars, 15 μm.
(PDF)

S5 Fig Statistical analysis of the interaction between genotype and time in Fig 5. Two-way ANOVA with Tukey’s post hoc analysis showed that there is a statistical interaction between genotype (control vs *Fbxl21* KO) and differentiation time (differentiation day 0, 2, 4, and 6) on *Myf5* mRNA expression, and protein expression of MYOGENIN and MYF5 (p < 0.05).
(PDF)

S6 Fig Diurnal expression pattern of MYOZ1 and FBXL21 observed in WT was abolished in *Psttm* mice. (A) Representative images of MYOZ1 staining in longitudinal sections of muscle tissues from WT and *Psttm* mice collected at ZT4 and ZT16. Muscles were longitudinally cryosectioned and stained with MYOZ1 antibody. Right panel: quantification of MYOZ1 expression. Data are presented as mean ± SEM (n = 3 mice/per group/time point). *p < 0.05, ***p < 0.001, and ****p < 0.0001; Two-way ANOVA with Tukey’s multiple comparisons. Scale bar: 2.5 μm. (B) Representative images of FBXL21 staining in the cross sections of the muscle tissues from WT and *Psttm* mice collected at ZT4 and ZT16. Muscles were cross-sectionally cryosectioned and stained with FBXL21 antibody. Right panel: quantification of FBXL21 expression. Data are presented as mean ± SEM (n = 3 mice/per group/time point). *p < 0.05 and ****p < 0.0001; Two-way ANOVA with Tukey’s multiple comparisons. Scale bar: 25 μm.
(PDF)

S1 Table JTK-Cycle analyses of *MyoD*, *Myogenin*, *Myf5*, and *Mrf4* in WT and *Psttm* mice in Fig 6D. (PDF)

S2 Table Sequences of qPCR primers used. (PDF)
