# Supplementary material for: The circadian E3 ligase FBXL21 regulates myoblast differentiation and sarcomere architecture via MYOZ1 ubiquitination and NFAT signaling
Source: PLoS Genet. 2022 Dec 27;18(12):e1010574. doi: 10.1371/journal.pgen.1010574 (PMC9829178; doi:10.1371/journal.pgen.1010574)
Supplement: S1 Fig — (A) MYOZ1 and FBXL21 show anti-phasic circadian oscillations in skeletal muscle. Lower panels: quantification of MYOZ1 and FBXL21 levels. Data are presented as mean ± SEM (n = 3 mice/time point). *p < 0.05; Two-way ANOVA shows a statistical difference between MYOZ1 and FBXL21. One-way ANOVA with Tukey’s post hoc analysis shows a statically significant differences in MYOZ1 amount between time points (ZT4 vs ZT16: *p < 0.05, ZT4 vs ZT20: *p < 0.05). (B) Co-localization of MYOZ1 and FBXL21 in 293T cells. 293T cells were transfected with indicated plasmids, and immunofluorescence staining was performed using the indicated antibodies. Scale bars, 15 μm. (PDF) [file pgen.1010574.s001.pdf]

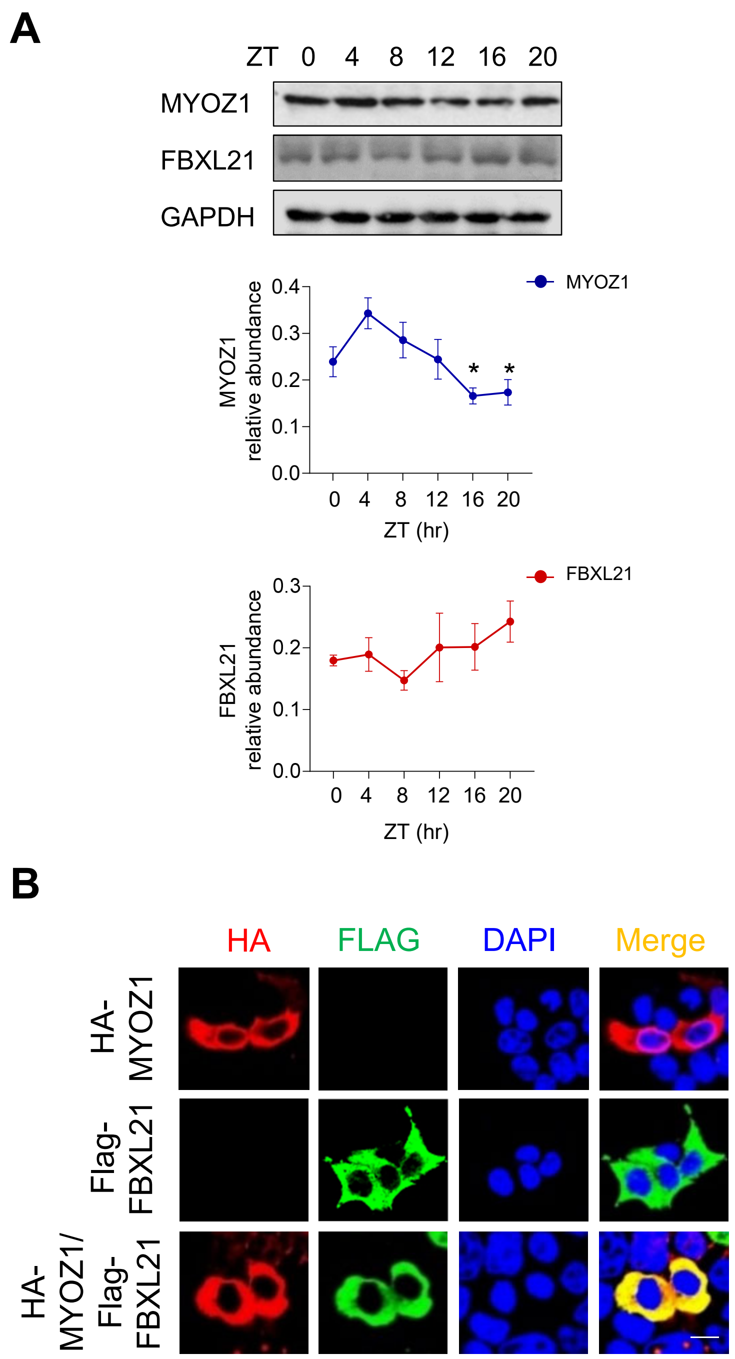

S1 Fig. Circadian rhythms and co-localization of MYOZ1 and FBXL21. (A) MYOZ1 and FBXL21 show anti-phasic circadian oscillations in skeletal muscle. Lower panels: quantification of MYOZ1 and FBXL21 levels. Data are presented as mean ± SEM (n = 3 mice/time point). *p < 0.05; Two-way ANOVA shows a statistical difference between MYOZ1 and FBXL21. One-way ANOVA with Tukey's post hoc analysis shows a statically significant differences in MYOZ1 amount between time points (ZT4 vs ZT16: *p < 0.05, ZT4 vs ZT20: *p < 0.05). (B) Co-localization of MYOZ1 and FBXL21 in 293T cells. 293T cells were transfected with indicated plasmids, and immunofluorescence staining was performed using the indicated antibodies. Scale bars, 15 μm.
