# Supplementary material for: The circadian E3 ligase FBXL21 regulates myoblast differentiation and sarcomere architecture via MYOZ1 ubiquitination and NFAT signaling
Source: PLoS Genet. 2022 Dec 27;18(12):e1010574. doi: 10.1371/journal.pgen.1010574 (PMC9829178; doi:10.1371/journal.pgen.1010574)
Supplement: S2 Fig — (A) FBXL21 protein expression in Fig 2C. (B) Protein expression of WT FBXL21 and FBXL21T33DS37D mutant in 293T cells in Fig 2D. 293T cells were transfected with the indicated plasmids. Cells were incubated with 100 μg/mL CHX for indicated time before harvest. (PDF) [file pgen.1010574.s002.pdf]

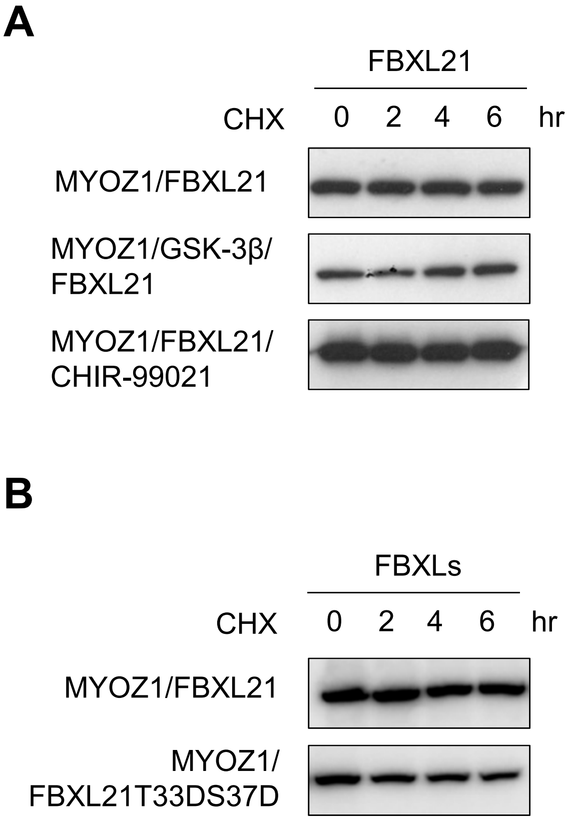

S2 Fig. Immunoblotting analysis of FBXLs expression in 293T cells. (A) FBXL21 protein expression in Fig 2C. (B) Protein expression of WT FBXL21 and FBXL21T33DS37D mutant in 293T cells in Fig 2D. 293T cells were transfected with the indicated plasmids. Cells were incubated with 100 μg/mL CHX for indicated time before harvest.
