# Supplementary material for: The circadian E3 ligase FBXL21 regulates myoblast differentiation and sarcomere architecture via MYOZ1 ubiquitination and NFAT signaling
Source: PLoS Genet. 2022 Dec 27;18(12):e1010574. doi: 10.1371/journal.pgen.1010574 (PMC9829178; doi:10.1371/journal.pgen.1010574)
Supplement: S3 Fig — (A) Loss of FBXL21 expression in Fbxl21 KO C2C12 cells compared to control cells. Successful Fbxl21 knockdown using the CRISPR-Cas9 was confirmed by immunofluorescence. Scale bars, 15 μm. (B) DAPI staining for Fig 3A. Scale bars, 15 μm. (C) Fusion index (%) quantification of Fig 3C. Control and Fbxl21 KO C2C12 cells were induced to differentiate for the indicated days (day 4 and day 6). The fusion index (%) was calculated. Data are presented as mean ± SEM (n = 3), ****p < 0.0001; Two-way ANOVA shows a statistical difference between control and Fbxl21 KO C2C12 cells. (PDF) [file pgen.1010574.s003.pdf]

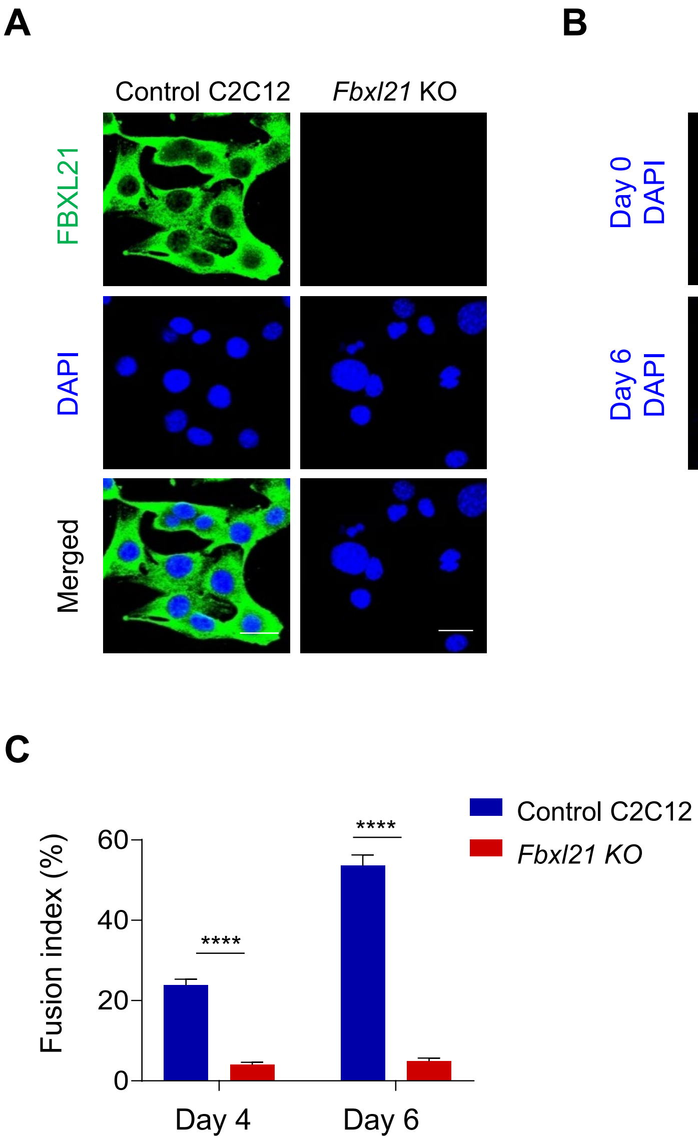

S3 Fig. Characterization and differentiation of *Fbxl21* KO C2C12 cells. (A) Loss of FBXL21 expression in *Fbxl21* KO C2C12 cells compared to control cells. Successful *Fbxl21* knockdown using the CRISPR-Cas9 was confirmed by immunofluorescence. Scale bars, 15 μm. (B) DAPI staining for Fig 3A. Scale bars, 15 μm. (C) Fusion index (%) quantification of Fig 3C. Control and *Fbxl21* KO C2C12 cells were induced to differentiate for the indicated days (day 4 and day 6). The fusion index (%) was calculated. Data are presented as mean ± SEM (n = 3), ****$p < 0.0001$; Two-way ANOVA shows a statistical difference between control and *Fbxl21* KO C2C12 cells.
