# Supplementary material for: The circadian E3 ligase FBXL21 regulates myoblast differentiation and sarcomere architecture via MYOZ1 ubiquitination and NFAT signaling
Source: PLoS Genet. 2022 Dec 27;18(12):e1010574. doi: 10.1371/journal.pgen.1010574 (PMC9829178; doi:10.1371/journal.pgen.1010574)
Supplement: S4 Fig — (A) qPCR of MyoZ1 mRNA in control and MyoZ1 KO C2C12 cells. Data are presented as mean ± SEM (n = 3), **p < 0.01; t-test. control vs. MyoZ1 KO C2C12 cells. (B) Representative MYOZ1 immunofluorescence staining image of successful MyoZ1 knockdown in C2C12 cells using CRISPR-Cas9. Scale bars, 15 μm. (C) Immunofluorescence staining of NFAT2 in control and MyoZ1 KO C2C12 myoblast cells. Control and MyoZ1 KO C2C12 myoblast cells were treated with DMSO or the calcineurin stimulators PMA (20 ng/ml) and ionomycin (0.25 μM) (PMA + Iono) for 1 hr. Right panel: quantification of % NFAT2 nuclear translocation. Data are presented as mean ± SEM (n = 4–6). Scale bars, 15 μm. ****p < 0.0001; t-test. Scale bars, 15 μm. (D) Immunofluorescence staining of MyHC in control and MyoZ1 KO C2C12 cells. Control and MyoZ1 KO C2C12 cells were differentiated for 4 days and stained with MyHC. Middle panel: MyHC-positive cells (%), right panel: fusion index (%) were calculated. Data are presented as mean ± SEM (n = 3). **p < 0.01 and ****p < 0.0001; t-test between control and MyoZ1 KO C2C12 cells. (E) Control and Fbxl21 KO C2C12 cells were treated with DMSO or the calcineurin stimulators PMA (20 ng/ml) and ionomycin (0.25 μM) (PMA + Iono) for 1 hr. Control and Fbxl21 KO C2C12 cells were immunostained for NFAT1 and imaged by confocal microscopy. In response to PMA and ionomycin treatment, NFAT1 nuclear translocation was increased in control C2C12 cells, while markedly decreased in Fbxl21 KO C2C12 cells. Right panel: quantification of NFAT1 nuclear translocation in control and Fbxl21 KO C2C12 cells. Data are presented as mean ± SEM (n = 3), *p < 0.05 and ****p < 0.0001; t-test. Scale bars, 15 μm. (PDF) [file pgen.1010574.s004.pdf]

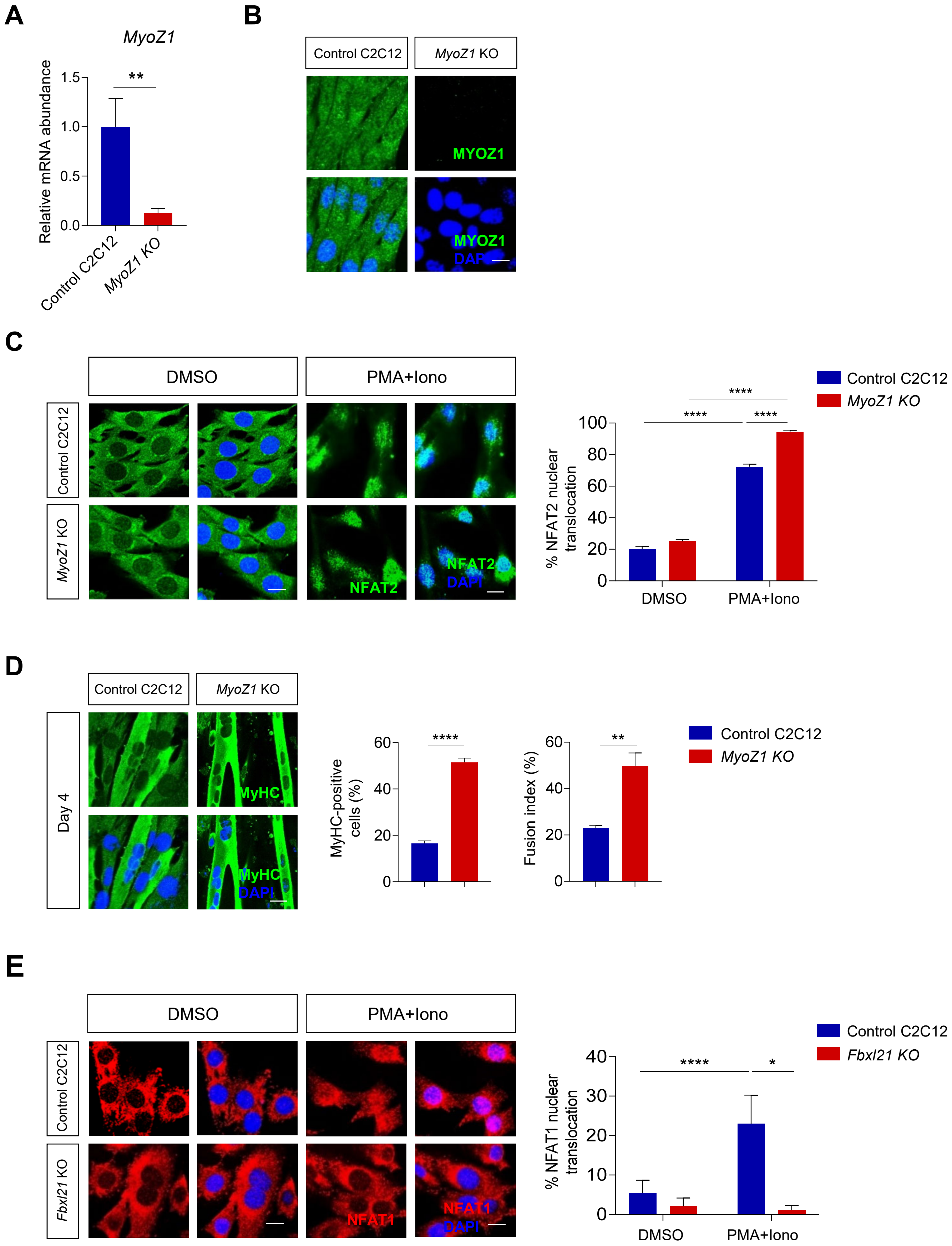
A
MyoZ1
Relative mRNA abundance
1.5
1.0
0.5
0.0
**
Control C2C12
MyoZ1 KO
B
Control C2C12
MyoZ1 KO
MYOZ1
MYOZ1
DAPI
C
DMSO
PMA+Iono
Control C2C12
MyoZ1 KO
NFAT2
NFAT2
DAPI
Control C2C12
MyoZ1 KO
% NFAT2 nuclear translocation
100
80
60
40
20
0
****
****
****
DMSO
PMA+Iono
D
Control C2C12
MyoZ1 KO
Day 4
MyHC
MyHC
DAPI
MyHC-positive cells (%)
60
40
20
0
****
Fusion index (%)
60
40
20
0
**
Control C2C12
MyoZ1 KO
E
DMSO
PMA+Iono
Control C2C12
Fbxl21 KO
NFAT1
NFAT1
DAPI
Control C2C12
Fbxl21 KO
% NFAT1 nuclear translocation
40
30
20
10
0
****
*
DMSO
PMA+Iono

**S4 Fig.** Altered NFAT localization in *MyoZ1* KO and *Fbxl21* KO C2C12 cells. (A) qPCR of *MyoZ1* mRNA in control and *MyoZ1* KO C2C12 cells. Data are presented as mean ± SEM (n = 3), **p < 0.01; t-test. control vs. *MyoZ1* KO C2C12 cells. (B) Representative MYOZ1 immunofluorescence staining image of successful *MyoZ1* knockdown in C2C12 cells using CRISPR-Cas9. Scale bars, 15 μm. (C) Immunofluorescence staining of NFAT2 in control and *MyoZ1* KO C2C12 myoblast cells. Control and *MyoZ1* KO C2C12 myoblast cells were treated with DMSO or the calcineurin stimulators PMA (20 ng/ml) and ionomycin (0.25 μM) (PMA + Iono) for 1 hr. Right panel: quantification of % NFAT2 nuclear translocation. Data are presented as mean ± SEM (n = 4 - 6). Scale bars, 15 μm. ****p < 0.0001; t-test. Scale bars, 15 μm. (D) Immunofluorescence staining of MyHC in control and *MyoZ1* KO C2C12 cells. Control and *MyoZ1* KO C2C12 cells were differentiated for 4 days and stained with MyHC. Middle panel: MyHC-positive cells (%), right panel: fusion index (%) were calculated. Data are presented as mean ± SEM (n = 3). **p < 0.01 and ****p < 0.0001; t-test between control and *MyoZ1* KO C2C12 cells. (E) Control and *Fbxl21* KO C2C12 cells were treated with DMSO or the calcineurin stimulators PMA (20 ng/ml) and ionomycin (0.25 μM) (PMA + Iono) for 1 hr. Control and *Fbxl21* KO C2C12 cells were immunostained for NFAT1 and imaged by confocal microscopy. In response to PMA and ionomycin treatment, NFAT1 nuclear translocation was increased in control C2C12 cells, while markedly decreased in *Fbxl21* KO C2C12 cells. Right panel: quantification of NFAT1 nuclear translocation in control and *Fbxl21* KO C2C12 cells. Data are presented as mean ± SEM (n = 3), *p < 0.05 and ****p < 0.0001; t-test. Scale bars, 15 μm.
