# Supplementary material for: The circadian E3 ligase FBXL21 regulates myoblast differentiation and sarcomere architecture via MYOZ1 ubiquitination and NFAT signaling
Source: PLoS Genet. 2022 Dec 27;18(12):e1010574. doi: 10.1371/journal.pgen.1010574 (PMC9829178; doi:10.1371/journal.pgen.1010574)
Supplement: S5 Fig — Two-way ANOVA with Tukey’s post hoc analysis showed that there is a statistical interaction between genotype (control vs Fbxl21 KO) and differentiation time (differentiation day 0, 2, 4, and 6) on Myf5 mRNA expression, and protein expression of MYOGENIN and MYF5 (p < 0.05). (PDF) [file pgen.1010574.s005.pdf]

| Figure | Gene names | P-value for interaction (genotype x time) |
|---|---|---|
| Fig 5A | *Myogenin* | $p = 0.23$ |
| | *MyoD* | $p = 0.53$ |
| | *Myf5* | $p < 0.01$ |
| | *Mrf4* | $p = 0.079$ |
| Fig 5B | MYOGENIN | $p < 0.0001$ |
| | MYOD | $p = 0.062$ |
| | MYF5 | $p < 0.05$ |

**S5 Fig.** Statistical analysis of the interaction between genotype and time in Fig 5. Two-way ANOVA with Tukey's post hoc analysis showed that there is a statistical interaction between genotype (control vs *Fbxl21* KO) and differentiation time (differentiation day 0, 2, 4, and 6) on *Myf5* mRNA expression, and protein expression of MYOGENIN and MYF5 ($p < 0.05$).
