# Supplementary material for: The circadian E3 ligase FBXL21 regulates myoblast differentiation and sarcomere architecture via MYOZ1 ubiquitination and NFAT signaling
Source: PLoS Genet. 2022 Dec 27;18(12):e1010574. doi: 10.1371/journal.pgen.1010574 (PMC9829178; doi:10.1371/journal.pgen.1010574)
Supplement: S6 Fig — (A) Representative images of MYOZ1 staining in longitudinal sections of muscle tissues from WT and Psttm mice collected at ZT4 and ZT16. Muscles were longitudinally cryosectioned and stained with MYOZ1 antibody. Right panel: quantification of MYOZ1 expression. Data are presented as mean ± SEM (n = 3 mice/per group/time point). *p < 0.05, ***p < 0.001, and ****p < 0.0001; Two-way ANOVA with Tukey’s multiple comparisons. Scale bar: 2.5 μm. (B) Representative images of FBXL21 staining in the cross sections of the muscle tissues from WT and Psttm mice collected at ZT4 and ZT16. Muscles were cross-sectionally cryosectioned and stained with FBXL21 antibody. Right panel: quantification of FBXL21 expression. Data are presented as mean ± SEM (n = 3 mice/per group/time point). *p < 0.05 and ****p < 0.0001; Two-way ANOVA with Tukey’s multiple comparisons. Scale bar: 25 μm. (PDF) [file pgen.1010574.s006.pdf]

A

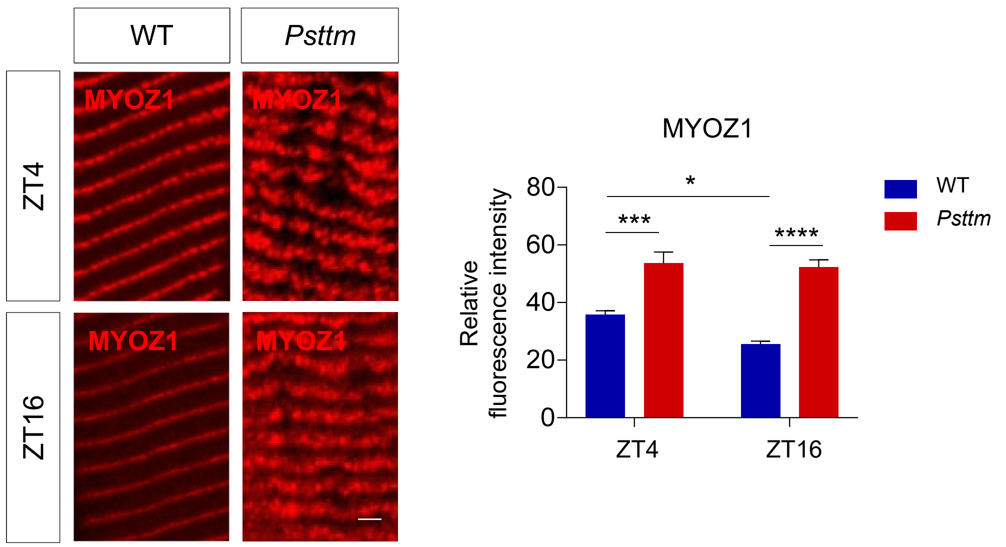

B

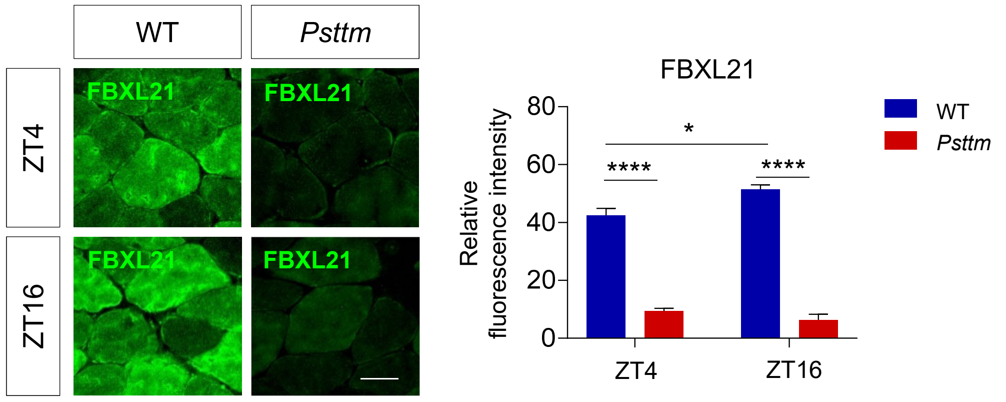

S6 Fig. Diurnal expression pattern of MYOZ1 and FBXL21 observed in WT was abolished in *Psttm* mice. (A) Representative images of MYOZ1 staining in longitudinal sections of muscle tissues from WT and *Psttm* mice collected at ZT4 and ZT16. Muscles were longitudinally cryosectioned and stained with MYOZ1 antibody. Right panel: quantification of MYOZ1 expression. Data are presented as mean ± SEM (n = 3 mice/per group/time point). *p < 0.05, ***p < 0.001, and ****p < 0.0001; Two-way ANOVA with Tukey's multiple comparisons. Scale bar: 2.5 µm. (B) Representative images of FBXL21 staining in the cross sections of the muscle tissues from WT and *Psttm* mice collected at ZT4 and ZT16. Muscles were cross-sectionally cryosectioned and stained with FBXL21 antibody. Right panel: quantification of FBXL21 expression. Data are presented as mean ± SEM (n = 3 mice/per group/time point). *p < 0.05 and ****p < 0.0001; Two-way ANOVA with Tukey's multiple comparisons. Scale bar: 25 µm.
