# Supplementary material for: The circadian E3 ligase FBXL21 regulates myoblast differentiation and sarcomere architecture via MYOZ1 ubiquitination and NFAT signaling
Source: PLoS Genet. 2022 Dec 27;18(12):e1010574. doi: 10.1371/journal.pgen.1010574 (PMC9829178; doi:10.1371/journal.pgen.1010574)
Supplement: S1 Table — (PDF) [file pgen.1010574.s007.pdf]

**S1 Table.** JTK-Cycle analyses of *MyoD*, *Myogenin*, *Myf5*, and *Mrf4* in WT and *Psttm* mice in Fig 6D.

| Group | Gene names | ADJ.P (adjusted *p*-value) | PER (period) | LAG (phase) | AMP (amplitude) |
|---|---|---|---|---|---|
| WT | *MyoD* | *P* < 0.001 | 20 | 12 | 0.12 |
| | *Myogenin* | *P* < 0.001 | 24 | 14 | 0.68 |
| | *Myf5* | *P* < 0.01 | 24 | 14 | 0.88 |
| | *Mrf4* | *P* = 0.21 | 24 | 14 | 3.1 |
| *Psttm* | *MyoD* | P = 0.062 | 8 | 2 | 0.042 |
| | *Myogenin* | P = 0.61 | 12 | 0 | 0.10 |
| | *Myf5* | P = 0.61 | 24 | 2 | 0.35 |
| | *Mrf4* | P = 0.51 | 24 | 8 | 1.28 |
