# Supplementary material for: The circadian E3 ligase FBXL21 regulates myoblast differentiation and sarcomere architecture via MYOZ1 ubiquitination and NFAT signaling
Source: PLoS Genet. 2022 Dec 27;18(12):e1010574. doi: 10.1371/journal.pgen.1010574 (PMC9829178; doi:10.1371/journal.pgen.1010574)
Supplement: S2 Table — (PDF) [file pgen.1010574.s008.pdf]

S2 Table. Sequences of qPCR primers used.

	Forward (5'-3')	Reverse (5'-3')
Myf5	CTGTCTGGTCCCAAAGAAC	TGGAGAGAGGGAAGCTGTGT
MyoD	CGGCATCTAGAGCCTGGTAG	CTGTCCTCAAAGCTGGGGTA
Myogenin	AGTGAATGCAACTCCCACAG	ACGATGGACGTAAGGGAGTG
Mrf4	GGGCCTCGTGATAACTGCTA	CCTGCTGGGTGAAGAATGTT
β-Actin	TTGTCCCCCCAACTTGATGT	CCTGGCTGCCTCAACACCT
Gapdh	CAAGGTCATCCATGACAACTTTG	GGCCATCCACAGTCTTCTGG
